# Hybridization in the wild between *Tursiops truncatus* (Montagu 1821) and *Delphinus delphis* (Linnaeus 1758)

**DOI:** 10.1371/journal.pone.0215020

**Published:** 2019-04-16

**Authors:** Rocío Espada, Liliana Olaya-Ponzone, Luisa Haasova, Estefanía Martín, José C. García-Gómez

**Affiliations:** 1 Laboratory of Marine Biology, Department of Zoology, Faculty of Biology, University of Seville, Seville, Spain; 2 Dolphin Adventure, Gibraltar, United Kingdom; 3 R+ D+I Biological Research Area, Seville Aquarium, Seville, Spain; 4 Research Foundation for University of Seville, (FIUS), Seville, Spain; University of Missouri Columbia, UNITED STATES

## Abstract

A case of intergeneric hybridization in the wild between a female bottlenose dolphin (*Tursiops truncatus*) and a short-beaked common dolphin (*Delphinus delphis*), considered members of *‘*vulnerable’ and ‘endangered’ subpopulations in the Mediterranean, respectively, by the International Union of Conservation of Nature is described in this paper. The birth of the hybrid was registered in the Bay of Algeciras (southern Spain) in August 2016, and the animal has been tracked on frequent trips aboard dolphin-watching platforms. This unique occurrence is the result of an apparent ongoing interaction (10 years) between a female bottlenose dolphin and common dolphins. The calf has a robust body with length similar to *Tursiops*, while its lateral striping and coloration are typical of *Delphinus*. It displays the common dolphin’s ‘criss-cross’ pattern. However, the thoracic patch is lighter than in *D*. *delphis* and its dorsal area is light grey, with a ‘V’ shape under the dorsal fin. This paper also provides a comprehensive mini-review of hybridizations of *T*. *truncatus* with other species.

## Introduction

**Fig 1:**
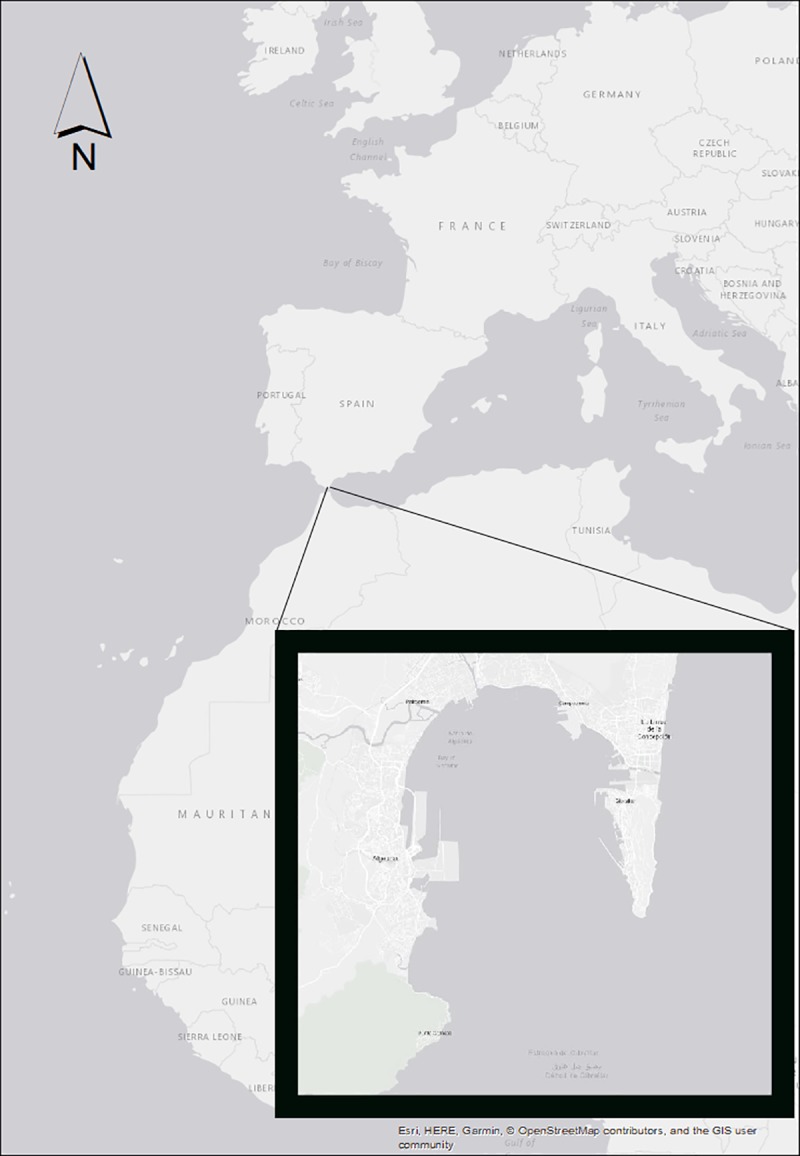
Study area, Bay of Algeciras. This map has been elaborated using GIS software ArcGIS 10.4.

The Bay of Algeciras, located in the south of Spain (Fig 1), hosts an important population of common dolphins (*Dephinus delphis*) which, since 2003, are considered ‘Endangered’ in the Mediterranean Sea according to the Red List criteria by the International Union of Conservation of Nature (IUCN) [1] and also ‘Vulnerable’ according to the Spanish National Catalogue of Endangered Species [2]. This area has been considered a feeding and breeding ground for this species [3, 4]. Also, it is possible to observe, more sporadically, groups of bottlenose dolphin (*Tursiops truncatus*), a species which is also considered as a ‘Vulnerable’ Mediterranean subpopulation by the IUCN. Striped dolphins *(Stenella coeruleoalba)* are occasionally detected (‘Vulnerable’ in the Mediterranean by IUCN) mixing with common dolphin, but the groups are mainly formed by mothers, calves and immature juveniles.

The three species involved in this study *D*. *delphis*, *S*. *coeruleoalba* and *T*. *truncatus*, included in the clade Delphininae [5], share the Bay of Algeciras in sympatric coexistence. They have a wide geographical distribution (the Atlantic, Pacific and Indian Oceans) and ‘can tolerate lower water temperatures and may occupy higher latitudes’ [6, 7]. Furthermore, it is known from fossil records that these species already belonged to three different genera in the Pleistocene, an epoch after which they may have diverged [8]. *T*. *truncatus* populations from the Black Sea (eastern Mediterranean) and Scotland (north-eastern Atlantic Ocean) [9] showed important genetic differences, which also exist between western Mediterranean and the Atlantic Ocean (Galicia and Portugal) ‘supporting evidences of a genetic boundary at the Almeria-Orán front’ [10]. Furthermore, genetic distinctions have been also detected between the Atlantic and Mediterranean stock in *S*. *coeruleoalba* and *D*. *delphis* species [11–16].

Common and bottlenose dolphins overlap ranges in temperate and tropical waters [17], although aggressive behaviours in bottlenose dolphins towards smaller species in different locations have been described [18]. According to observations in the Bay of Algeciras, mixed groups of both species have never before been recorded locally. However, an ongoing intergeneric interaction between a lone female bottlenose dolphin, commonly known as ‘Billie’ and groups of common dolphins has been observed since 2006.

The bottlenose dolphin was identified among the common dolphins due to morphologic differences in size and coloration: she was bigger than the common dolphins, with a robust body, a falcate dorsal fin, of light grey coloration to darker grey dorsally, and showed a light blaze marking on her sides. A well marked demarcation at the end of the melon, convex flippers and a short and stubby beak [17] were also recognisable features in the individual. The gender (female) was supported by photographic evidence.

Before 2016, Billie was detected three times assisting common dolphin births, leading newborns to the surface and offering alloparental care for only a few minutes after labour and always accompanied by other common dolphins. Allomaternal care often occurs among bottlenose dolphins [19, 20] and has been described in captivity and in the wild [21, 22]. However, it is uncommon to observe a bottlenose dolphin calf adopted by a common dolphin, although it has been previously reported in the northern Adriatic waters [23].

**Fig 2:**
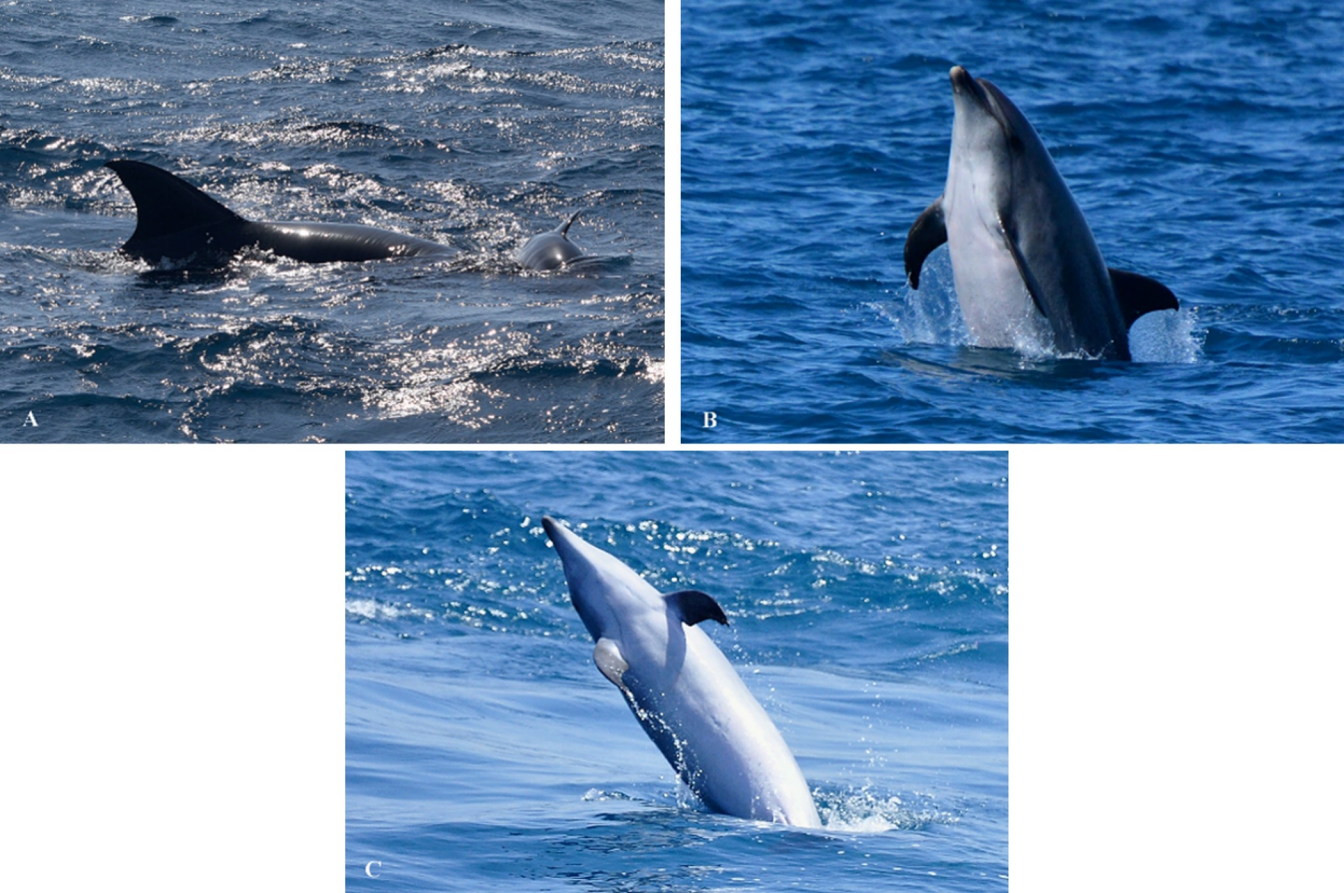
Billie raising the newborn to the surface, and comparison of the bodyshape of the pregnant/not pregnant female bottlenose dolphin. (A) On 11 August the female bottlenose dolphin was observed holding and pushing a newborn to the surface. The newborn showed folded fins and marked foetal folds. (B) Billie pregnant (28 July 2016). (C) Billie not pregnant (27 May 2017).

On 11 August 2016, Billie was observed raising and pushing to the surface a neonate (Fig 2A). Visible features such as foetal folds (vertical depigmented lines) were visible on each flank of the individual, as well as a bent dorsal fins and curled flukes, produced by the neonatal posture in the uterus during the gestation period [24]. No other dolphins were present. In view thereof, and taking into account the observations mentioned above, a hybridization was considered. It was possible to compare photographs before and after Billie´s pregnancy, showing weight gain and grosser bodyshape while pregnant (Fig 2B). Billie showed a much slimmer and thinner bodyshape when she was not pregnant (Fig 2C).

Cetaceans exhibit surprising karyotype uniformity, suggesting that they have a higher potential to produce hybrid offspring than do other mammals [25–27]. However, the identification of cetacean hybrids in the wild is difficult, and the molecular evidence of wild cetacean hybrids is extremely limited [28]. This is not to say that these hybridizations in mammals are uncommon and do not occur in various marine and terrestrial species [29–31].

Marine mammal hybridizations are very difficult to detect in comparison to other taxonomic groups of animals and plants, although several examples in captivity and in the wild have been reported [32–35]. Intergeneric or interspecific reproduction is more likely to happen between species when their habitat ranges overlap, when different species have the same habits or similar social behaviours. Hybridization can also be a result of particular and unique life histories developed within dolphin groups [36], as had resulted in this particular case, where a single female bottlenose dolphin had been adopted by groups of common dolphins.

## Methods

The study was carried out on board opportunistic dolphin-watching platforms (14 m and 12 m in length), offering trips of 90 min with consistent daily itineraries from August 2016 until May 2017. Once the group of interest (the one including the mother/hybrid pair) was detected, standardized data were gathered (weather permitting), such as date, time, GPS position, structure and group size. Group composition data were collected using a combination of sampling methods. Individual-following protocols [37, 38], focusing on the mother/hybrid pair were applied during the sightings. The 10 m chain rule [39] was also applied: the pair were determined to be together if they were less than 10 m apart. Swimming positions, general behaviour and body-contact events were also gathered when they were displayed, irrespective of the time. Sea surface temperature was measured from the side of the boat, using a digital thermometer with 0.1°C graduations [40]. The mother/hybrid pair and other dolphins were photographed (Nikon DSLR camera, Nikon 70–300 mm lens) for re-identification and also for morphological analyses of the presumed hybrid [41]. In some cases, images were slightly retouched (descriptors: saturation, contrast, exposition, clearness and shades), with Adobe Photoshop Lightroom software, to improve the display of the morphological features described in the text. A plotter map was elaborated using ArcGIS 10.4 software, including coordinates of the mother/hybrid pair.

Tissue sampling by means of biopsy dart was not attempted as it was considered invasive and inappropriate due to the immaturity of the calf [42]. It is well known that newborns obtain temporary immunological protection from maternal antibodies, and the immune system of many mammalian species is not fully developed at birth [43]. Skin swabbing [44] was also considered for genetic analysis, but cautious measures were taken, ensuring that the calf was at least one year of age before the tests were attempted.

Data regarding the group composition of the species involved were collected over 25 weeks (11 August 2016 to 29 May 2017). Sightings were classified according to 19 descriptors. A box plot analysis (using SPSS 15 statistical software IBM, New York, NY, USA), was applied to analyse the behaviour of the mother/hybrid pair, comparing the composition and the frequency they were found in mixed-species groups, separated or alone.

This study was carried out in strict accordance with the cetacean protocol included in the Marine Regulations, 2014 and has been approved by the Committee on the Ethics of Animal Experiments of the Ministry for Education, Heritage, Environment, Energy and Climate Change of Gibraltar.

## Results

**Fig 3:**
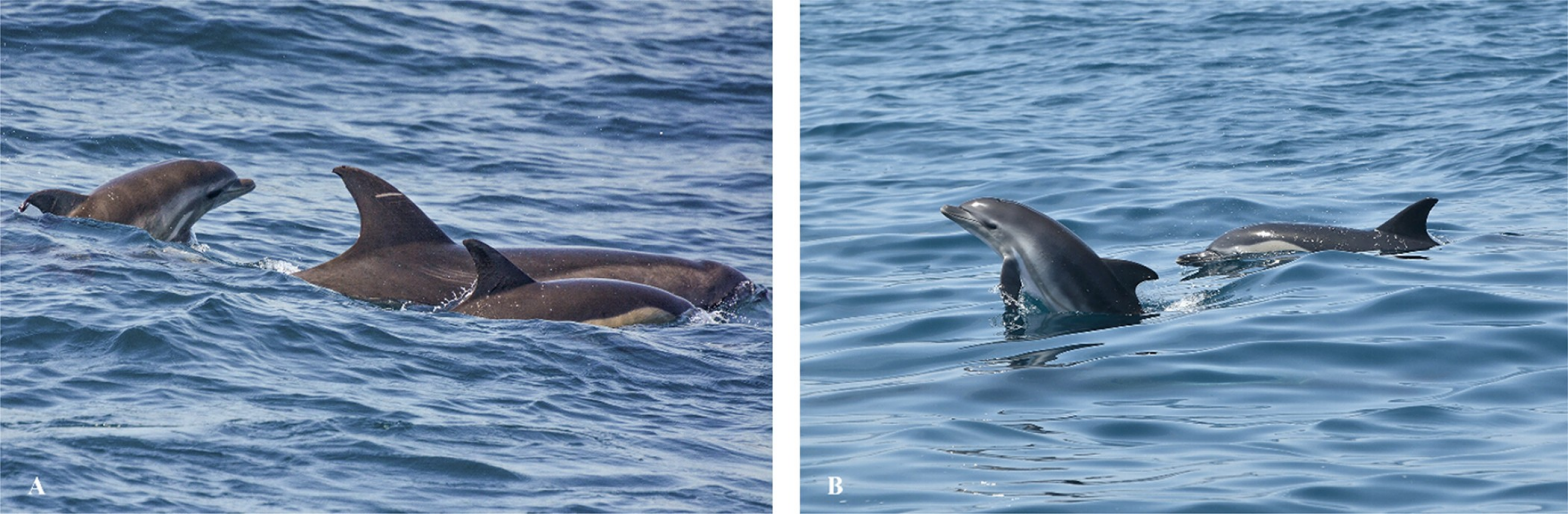
Mixed group of mother/hybrid pair with common dolphins. (A) The mother/hybrid pair and an adult common dolphin spotted on 6 October 2016; (B) Hybrid displaying jumps alongside a common dolphin separated by more than 10 m from the presumed mother on 4 September 2016.

After the first sighting on 11 August 2016, re-sighting took place on 17 August 2016, after which they were seen on an almost daily basis mixing with ‘nursery groups’ of common dolphins (*D*. *delphis*) (Fig 3A and 3B). Data were collected between 17 August 2016 and 4 June 2017. The pair was observed 113 times (57 h 11 min of observation) in a total of 355 sightings. Of these, 104 times (53 h 55 min) the pair was found within nursery groups of common dolphins formed by females and calves [45], twice (1 h 23 min) in mixed nursery groups of common dolphins accompanied by mothers, calves and immature juveniles of striped dolphins *(S*. *coeruleoalba)* and in only six sightings (1 h 53 min) was the pair sighted alone, distanced from the common dolphins (minimum 500 m between groups). The pair were detected together less than 10 m apart in 112 sightings (99.1%); 1 occasion (0.83%) was Billie (female *T*. *truncatus*) separated from the hybrid by 100 m, both of them accompanied by common dolphins. Sea surface temperature (SST) during these observations was an average of 19.35°C (66.83°F) with minimum of 14°C (57.2°F) and maximum of 26°C (78.8°F). From 2 June 2017 until the end of the year, the hybrid was not sighted again, leading to the reasonable suspicion of death.

Table 1 presents every sighting recorded during the campaign classified according to the group composition represented by 19 different descriptors, depending whether the mother/hybrid pair was separated, together, alone or mixed with other species.

**Table 1:** Data regarding species group composition collected over 25 weeks (period 11 August 2016 to 29 May 2017). Sightings were classified according to 19 descriptors: (A1) Common dolphins only; (A2) Striped dolphins only; (A3) Bottlenose dolphins only; (B1) Common dolphins with mother/hybrid pair together; (B2) Striped dolphins with mother/hybrid pair together; (B3) Bottlenose dolphins with mother/hybrid pair together; (C1) Common dolphins with mother/hybrid pair not together; (C2) Striped dolphins with mother/hybrid pair not together; (C3) Bottlenose dolphins with mother/hybrid pair not together; (D1) Common dolphins with one of the mother/hybrid pair (mother or hybrid); (D2) Striped dolphins with one of the mother/hybrid pair (mother or hybrid); (D3) Bottlenose dolphins with one of the mother/hybrid pair (mother or hybrid); (E1) Mother/hybrid pair only; (E2) Mother only; (E3) Hybrid only; (F1) Common mixed with striped dolphins; (F2) Common mixed with bottlenose dolphins; (F3) Bottlenose mixed with striped dolphins; (F4) Common and striped dolphins mixed with mother/hybrid pair.

Week	A1	A2	A3	B1	B2	B3	C1	C2	C3	D1	D2	D3	E1	E2	E3	F1	F2	F3	F4
**1**	1,286	0,143	0,571	1,286	0	0	0	0	0	0	0	0	0	0	0	0	0	0	0
**2**	2,4286	0	0	2,286	0	0	0	0	0	0	0	0	0	0	0,286	0	0	0	0
**3**	2	0	0,2	1,2	0	0	0	0	0	0,2	0	0	0,2	0	0	0,2	0	0	0
**4**	2,667	0	0,667	1,667	0	0	0	0	0	0	0	0	0,167	0	0	0	0	0	0
**5**	2,333	0	0	1	0	0	0	0	0	0	0	0	0	0	0	0,667	0	0	0
**6**	2	0	0	1,143	0	0	0	0	0	0	0	0	0,167	1,423	0	0	0	0	0
**7**	1,5	0	0	0,667	0	0	0	0	0	0	0	0	0	0	0	0,333	0	0	0
**8**	1,333	0	0,167	1,333	0	0	0	0	0	0	0	0	0	0	0	0	0	0	0,167
**9**	2	1,1667	0,1667	0,833	0	0	0	0	0	0	0	0	0,167	0	0	1,167	0	0	0
**10**	1,833	0,333	1	0,833	0	0	0	0	0	0	0	0	0	0	0	0,167	0	0	0
**11**	1,333	0	0,5	0,167	0	0	0	0	0	0	0	0	0,167	0	0	0,167	0	0	0,167
**12**	1,143	0	0,286	0,571	0	0	0	0	0	0	0	0	0	0	0	0	0	0	0
**13**	1	0	0	0,833	0	0	0	0	0	0	0	0	0	0	0	0	0	0	0
**14**	1	0	0	1,2	0	0	0	0	0	0	0	0	0	0	0	0	0	0	0
**15**	1,25	0,25	0	0,75	0	0	0	0	0	0	0	0	0	0	0	0	0	0	0
**16**	1	0	0	0,6	0	0	0	0	0	0	0	0	0	0	0	0	0	0	0
**17**	1	0	0	0,667	0	0	0	0	0	0	0	0	0	0	0	0	0	0	0
**18**	1	0	0	0,25	0	0	0,25	0	0	0	0	0	0	0	0	0,25	0	0	0
**19**	0,75	0,25	0	1	0	0	0	0	0	0	0	0	0	0	0	0	0	0	0
**20**	0,667	0	0	0,333	0	0	0	0	0	0	0	0	0,333	0	0	0	0	0	0
**21**	0,571	0,714	0,143	0	0	0	0	0	0	0	0	0	0	0	0	0,143	0	0	0
**22**	1,5	0,25	0,5	0	0	0	0	0	0	0	0	0	0	0	0	0	0	0	0
**23**	3	0	0,25	0	0	0	0	0	0	1,5	0	0	0	0	0	0	0	0	0
**MEAN**	1,5041130	0,1350739	0,193508	0,8095217	0	0	0,0108695	0	0	0,0739130	0	0	0,0522173	0,0618695	0,0124347	0,1345217	0	0	0,014521
**SD**	0,6636513	0,2836602	0,275524	0,5628901	0	0	0,052128	0	0	0,3136549	0	0	0,0952823	0,2967160	0,0596351	0,2763548	0	0	0,048113

The female bottlenose dolphin was observed showing continuous epimeletic and nurturant behaviour towards the newborn, offering care and protection and exhibiting near-body contact for the first three months of observation. The two main swimming positions for calves and their mothers are defined as ‘echelon position’ (the calf swimming alongside the mother) and ‘infant position’ (the calf swimming under the mother) [19]. The hybrid was observed in the echelon position most of the time; in the infant position on only two occasions, when the pair approached to bow-ride during the study period. At this time, the young calf still showed clear foetal folds [46].

Calves often show rubbing behaviour with their mothers, with particular focus on her head region [19]. Body-contact events such as flipper–belly, flipper–flipper, forehead–belly, head and beak rubbing, blowhole rubbing, back-to-back calf jumping backwards over the mother’s back) and petting were observed between Billie and the hybrid, and were considered typical behaviour displays between a mother and her calf [47, 48]. Mother chasing towards the newborn and vice versa were also detected, which is shown to be indicative of an imprinting period [19]. When the calf´s rostrum was in contact with the mother’s mammary slit area for longer than 2 s [19] it was recognised as a nursing event and was recorded at least four times on 19, 23 and 27 August 2016 and 2 September 2016.

The mother/hybrid pair showed normal breathing and developmental patterns and close swimming positions until 4 September 2016, when at 24 days the neonate was observed breaching and swimming in echelon position alongside an adult common dolphin (Fig 3B). At the time Billie was observed displaying feeding behaviour among other common dolphins. After this event, the calf returned to its mother’s side.

From photographs, morphological features of the presumed hybrid offspring were compared with both common dolphin (*D*. *delphis*) and bottlenose dolphin (*T*. *truncatus*) (Table 2), and characteristics of both species were confirmed in the neonate. In addition, no mammary slits were observed, and a separation between the genital and anal slits was documented, suggesting the neonate was male [49].

**Table 2:** Comparison of morphological features between species. Morphological features of common dolphins (*D*. *delphis*), bottlenose dolphin (*T*. *truncatus*) (Mediterranean population) and the presumed hybrid, recorded in the Bay of Algeciras, south of Spain.

	*Delphinus delphis*	*Tursiops truncatus*	Presumed Hybrid
Size at birth	0.76–0.86 m [50]	0.9–1.3 m, 32 kg [50]	1.25 m (Estimated)
Body shape	Slender [51]	Robust [51]	Robust [51]
Beak	Long beak sharply demarcated from the melon [52]	Rounded forehead/ marked creased beak. Short and stubby beak [52]	Rounded forehead/ marked creased, short and stubby beak [35]
Body colour pattern	Criss-cross pattern; brownish/black back, ‘V’ shape under dorsal fin; yellowish thoracic patch; light grey posterior patch; white belly. [51, 52]	Light grey to black dorsally and laterally; light belly; light blaze or brush marking sometimes observed on their flanks.[51, 52]	Medium/dark grey back; pale creamy flanks from eye to peduncle; white belly and post-pectoral patches.
	Dark flipper-to-anus stripe parallel to the lower margin of the cape; dark flipper stripe joining the lip patch on the underside of the beak. [35–51]		Light creamy yellow/greyish stripe from the back of the eye to its posterior flanks.
Dorsal fin	Tall/moderate falcate	Falcate	Falcate
	Clear patch sometimes [35, 51]	Dark. [35, 51]	Dark
Mouth-to-flipper stripe	Present	Absent	Present

According to Whitehead and Mann [53], *Tursiops spp*. Neonates are 1.1 m, while in common dolphins 0.8 m. The presumed hybrid was less than half the size of the female bottlenose at birth (approximately 1.25 m), and remained in echelon position close to her most of the time, characteristics consistent with a newborn. By the middle of November, the animal was just over half the length of Billie, and therefore considered an infant or calf [19, 38, 40]. The neonate’s beak was short and stubby, with a round melon and robust body, showing more similarities to the bodyshape of a bottlenose dolphin than to that of a common dolphin [24, 40, 51].

**Fig 4:**
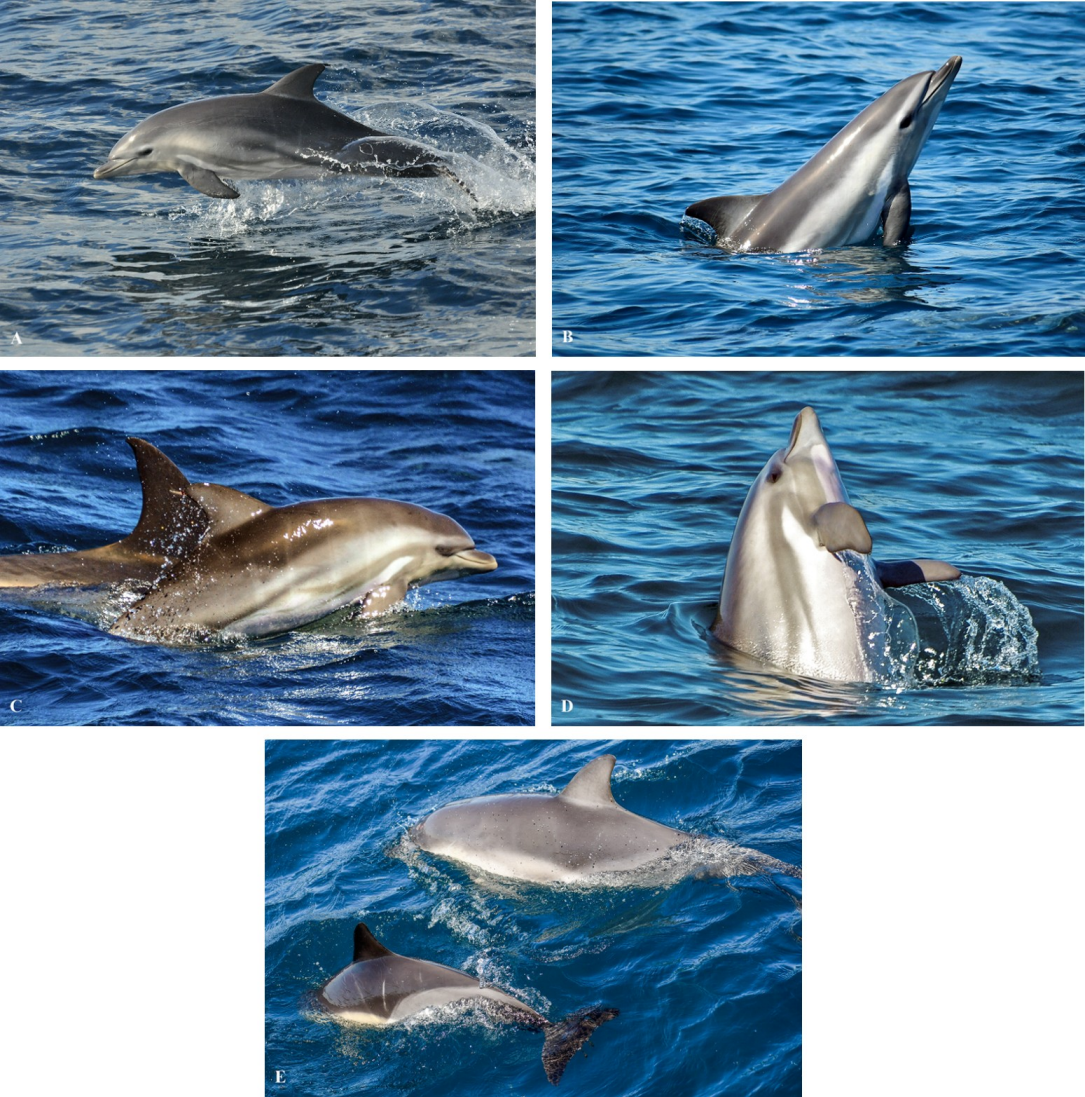
Morphological features as coloration and shape patterns of the presumed hybrid. (A) muted ‘criss-cross’ pattern typical of common dolphins, photographed 5 November 2016. (B) Creamy yellow patch on its side documented 7 February 2017; (C) light grey posterior flank on 20 February 2017; (D) Striped pattern on 20 February 2017; (E) Comparison between potential hybrid (top right) and common dolphin calf (bottom left). A similar ‘V’ shape and light-coloured dorsal fin can be observed in these dolphins. Documented on 20 February 2017.

**Fig 5:**
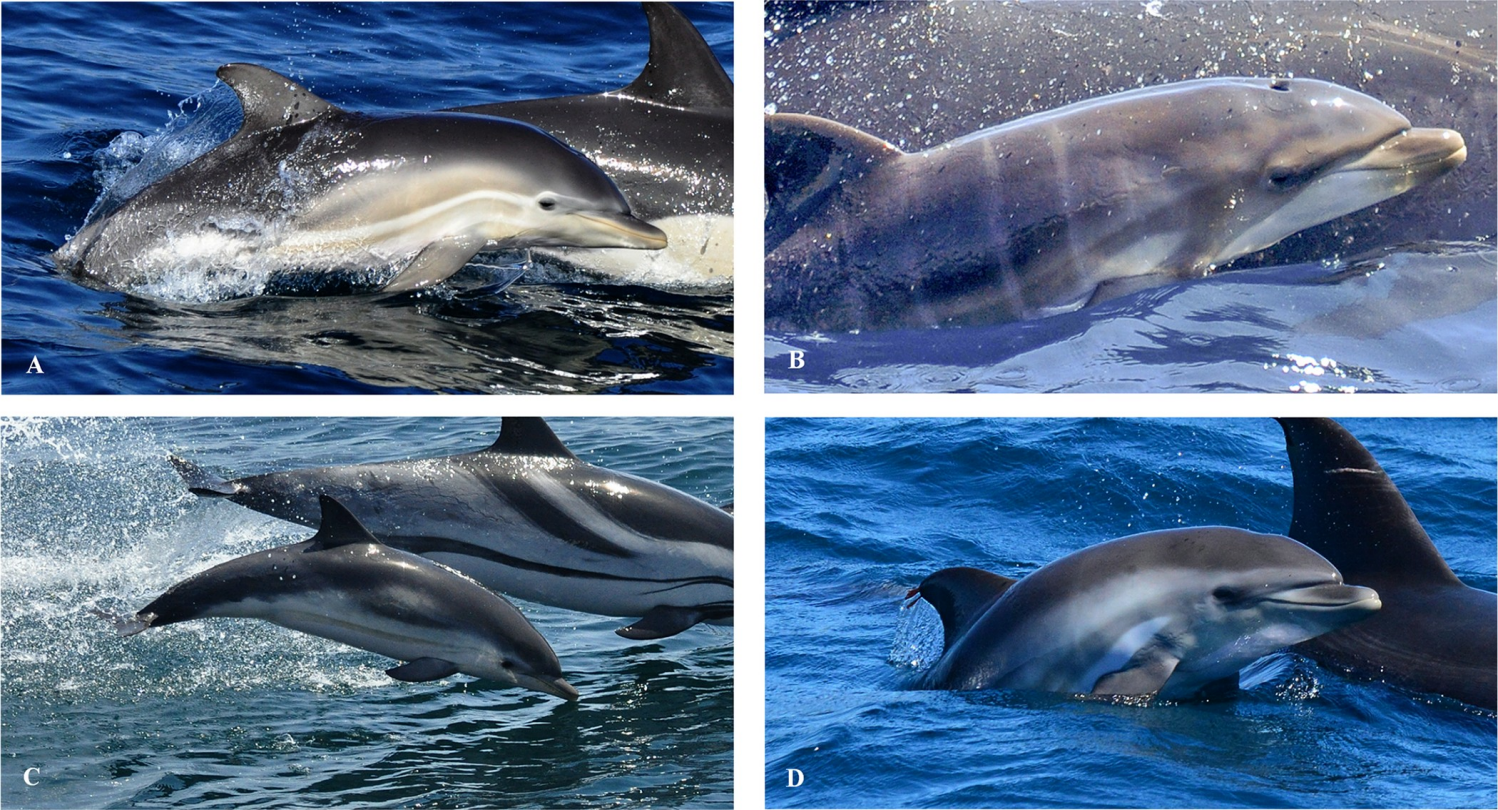
Coloration features displayed by *D*. *delphis*, *T*. *truncatus*, *S*. *coeruleoalba* and the hybrid calves. Notice the similarities of colour patterns between (A) *D*. *delphis*, (B) *T*. *truncatus* and the hybrid (D). These similarities are absent when comparing features of (C) *S*. *coeruleoalba* (bluish-grey dorsally, white to light grey blaze on the flanks, eye-to-anus stripe that runs ventrally [55]) and the hybrid.

Coloration and striping patterns were examined from photographs. A typical ‘criss-cross’ coloration along the neonate’s thorax and flank was detected, corresponding to markings characteristic of common dolphins (Fig 4A). A pale creamy-coloured patch ran from the low melon/rostrum/eye along the thorax, which faded at the light grey posterior flank patch (Fig 4B and 4C). The hybrid had a flipper stripe, which is characteristic of *D*. *delphis*, although in this case it was light brown in colour from the anterior insertion of the flipper to the lower jaw and gape (Fig 4D). Two other stripes were identified: one that ran from the caudal canthus of the eye to the anterior insertion of the flipper and a second above the flipper stripe, from the caudal canthus of the eye to its flank. Both were slightly darker in colour (Fig 4C and 4D). Also, it showed a white patch between the dorsal and ventral stripe (Fig 4C and 4D), which has also been observed in other bottlenose dolphins neonates in the bay (Fig 5A–5D). The neonate’s sides were light grey, with a V-shaped pattern on its side under the dorsal fin. The dorsal fin was bigger and wider than in common dolphins and grey, becoming much lighter over time (Fig 4E). The ventral side was white.

Dolphin neonates show lines across their flanks and backs called foetal folds [54]. By 21 December 2016, the hybrid no longer showed foetal folds so it was considered an infant.

**Fig 6:**
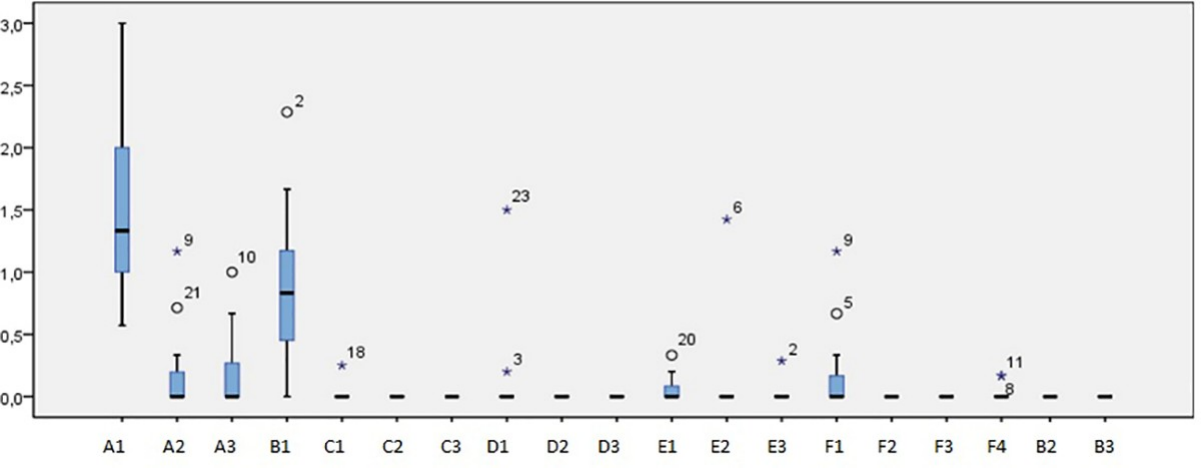
Box plot analysis exposing medians, quartiles and confidence interval bars (95%) of data reported in Table 1, referred to 19 types (descriptors A1, A2 …F4) of sighting. The extreme values (asterisks) are those that were more than three times the interquartile range from Q3. Outliers (white circles) are those that were located between 1.5 and 3 times the interquartile range from Q3. The figure clearly shows the close relationship of Billie and the newborn hybrid with common dolphins (B1), their relationships being completely nil with striped dolphins (A2) and with bottlenose dolphins (B3). The number of sightings of Billie and the newborn hybrid together in the absence of other dolphins also deserves mention (E1).

Data analysis from Table 1 is represented in Fig 6. Only the descriptors A1, A2, A3, B1, E1 and F1 resulted in valuable information, thus making it possible to compute the median (robust measure of central tendency, independent from the extreme scores). The other variables registered exceptional or no sightings. Groups of exclusively common dolphins (A1) were those most often spotted in the area. When the mother/hybrid pair were detected included in mixed-species groups, this was mostly with nursery groups of common dolphins (B1). A third species (striped dolphin) was located in the mixed groups on an exceptional basis, but they always proved to be mothers, with immature juveniles and calves (F4). The pair was detected alone (E1) on a few occasions, at a distance of more than 500m from the groups of common dolphin.

## Discussion

There is little information about hybrids in the wild; therefore, the significance of this potential hybridization is two-fold. First, this event between these species in the wild supports what has been observed in the non-natural conditions of captivity. Second, species such as *T*. *truncatus* and *D*. *delphis*, with spatially overlapping habitats [17], have rarely been recognised as interbreeding until now. Moreover, this type of intergeneric interaction occurs at a low level, as although the habitats of the two species described overlap, they rarely mix.

On the other hand, hybridization events in Delphinidae in captivity have been reported multiple times, and *T*. *truncatus* hybrids have been described interbreeding with several species (Table 3), but this event (*Stenella frontalis* × *T*. *truncatus*) has only been observed once in the wild [56, 57]. In captivity, intergeneric hybridization was produced by a cross between *T*. *truncatus* and several other species including *Delphinus capensis* [35, 58], which resulted in four hybrids. Two of the calves died, but a living fertile female back-crossed with a *T*.*truncatus*, and the calf didn’t survive either. In 2018, Gridley, reported multiple intra-generic matings between *T*. *truncatus* and *Tursiops aduncus* producing a health F_1_ hybrid, which survived to adulthood and also produced back-crossed hybrid offspring [59].

**Table 3:** Registry table of hybridization between individuals of bottlenose dolphins (*T*. *truncatus*) in captivity and in the wild. Adapted and expanded from [60] and [61].

Parental species	Number of hybrids F_1_	References	Environment
*T*. *truncatus* x *Grampus griseus*	3	[62]	Captivity
*Globicephala macrorhynchus* x *T*. *truncatus*	2	[58, 63]	Captivity
*Steno bredanensis* x *T*. *truncatus*	1	[64]	Captivity
*T*. *truncatus* x *Pseudorca*. *Crassidens*	6	[58, 65]	Captivity
*Llagenorhynchus obliquidens* x *T*. *truncatus*	1	[66]	Captivity
*T*. *truncatus* x *G*. *griseus*	13	[66, 67, 68, 69]	Captivity
*T*. *truncatus* x *S*. *frontalis*	1	[56, 57]	Wild
*T*. *truncatus* x *D*. *capensis*	4	[35]	Captivity
*S*. *guianensis* x *T*. *truncatus*	1	[70]	Captivity
*T*. *truncatus x T*. *aduncus*	7	[59]	Captivity
*T*. *truncatus x D*. *delphis*	1	This paper	Wild

According to morphological [71, 52] and genetic [72, 73] studies, *S*. *coeruleoalba* and *Delphinus* have a closer phylogenetic relationship, being more closely related to each other than to *T*. *truncatus*. Furthermore, the greatest number of interactions between the three species cited have been observed between *D*. *delphis* and *S*. *coeruleoalba* [74–81]. In fact, *D*. *delphis* and *S*. *coeruleoalba* coexist in sympatry in three different areas of the Mediterranean, including the Alborán sea [79–81]. In addition, ‘*S*. *coeruleoalba* displayed more opportunistic trophic habits compared with *D*. *delphis*’ in the north of Spain (Bay of Biscay) [78]. ‘Fission-fusion grouping patterns’ have been described between *T*. *truncatus* and *D*. *delphis* [82, 83], depending on the distribution and availability of food sources. Furthermore, in the eastern Ionian Sea, when both species coexist in ‘direct sympatry’ [81], habitat partition results [82, 84]. A niche separation has been suggested that might have reduced the direct food-base competition [82] in such species, observing ‘different foraging strategies, with *D*. *delphis* feeding in the water column or near the surface and *T*. *truncatus* focusing on bottom prey’ [85]. This has been also observed in the Bay of Algeciras.

Accordingly, the probability of hybridization of *D*. *delphis* with *S*. *coeruleoalba* was expected to be higher than with *T*. *truncatus*. However, interactions between *D*. *delphis* and *T*. *truncatus* [82, 86] are well known, as are sympatric associations between the species, and according to the sympatry concept [81], ‘the co-occurrence of two or more dolphin species in the same immediate habitat’ [81, 82, 86] can increase the possibility of hybridization. Also to be taken in consideration is the high level of promiscuity of *T*. *truncatus* and their potentiality to produce hybrids with up to ten different genera of dephinids (Table 3). All factors mentioned above strongly support that the hybrid described in this paper is the result of at least 10 years of integration of Billie into groups of. *D*. *delphis*. This is corroborated in Table 1, which shows that Billie was mixing to a negiglible degree with mothers and sexually immature *S*. *coeruleoalba* calves.

Despite the uniqueness of this hybridization, DNA samples from the hybrid were not obtained. Considerations were that the rare, but extremely dangerous experiences during cetacean sampling [87, 88], the death of a common dolphin while being sampled by a dart [89], and, most importantly, the early and delicate developmental stage of the calf, made taking a biopsy too risky.

The Bay of Algeciras is a heavily anthropised area, but it serves as a feeding, nursing and breeding ground for cetaceans, including both common and bottlenose dolphins and future hybrids. Enforcement of the cetacean observation protocols and the introduction of an environmental education plan to minimise the impacts on cetaceans in the Bay of Algeciras are vital. In this regard, conservation measures have already been proposed for this hotspot area for cetaceans facing detrimental threats [4].
